# Essential Attributes of a Successful Dentist: A Quantitative Study of Dental Students at a University in Mainland China

**DOI:** 10.3390/dj14020107

**Published:** 2026-02-12

**Authors:** Jasmine Cheuk Ying Ho, Hollis Haotian Chai, Michelle Zeping Huang, Edward Chin Man Lo, Hao Yu, Chun Hung Chu

**Affiliations:** 1 Faculty of Dentistry, University of Hong Kong, Hong Kong 852, Chinahchai89@hku.hk (H.H.C.); michellehuang@hsu.edu.hk (M.Z.H.);; 2 Department of English, The Hang Seng University of Hong Kong, Hong Kong 852, China; 3 School of Stomatology, Fujian Medical University, Fuzhou 350000, China

**Keywords:** dental practice, practice management, satisfaction, dental education

## Abstract

**Background**: The pursuit of a successful dental career extends beyond clinical skills to encompass various personal and professional attributes. Understanding the qualities that define a successful dentist from the perspective of dental students is essential. **Objective**: This study aims to explore the perspectives of dental students at a university in mainland China regarding the attributes that define a good or successful dentist. **Methods**: A cross-sectional survey was administered to dental students at Fujian Medical University (FJMU) from March to August 2025 using an anonymous questionnaire. The questionnaire included four self-administered questions: (1) qualities associated with “a successful dentist”, (2) qualities of “a good dentist”, (3) qualities students expect from their own dentists, and (4) qualities dental education should emphasize. For each question, participants selected the three most important attributes from a list of 23 predefined options or provided their own responses. **Results**: All 651 dental students at FJMU were invited to participate, with 645 (99%) completing the survey. Their ages ranged from 18 to 24 years, and 281 (44%) were male. Clinical competence, experience, knowledge, good communication skills, and a sense of responsibility or accountability were consistently ranked as the top five attributes by dental students across all four survey questions. Attributes such as punctuality and altruism received the lowest ratings. Bivariate analysis revealed that senior students (Years 4–5) considered good communication skills a more important quality than junior students (Years 1–3) across all four questions. **Conclusions:** Dental students in mainland China perceive clinical competence, experience, and knowledge as essential attributes for dental practice, alongside communication skills and responsibility.

## 1. Introduction

A successful dental career is about more than just clinical expertise. Dentists need a combination of personal and professional qualities. Dental students’ ideas about what makes a “good” or “successful” dentist are important. These beliefs shape their education, how they grow as professionals, and how they will treat patients in the future. Despite the critical need for conceptual clarity, the dental literature lacks consensus on defining these terms, with no universally accepted framework for essential qualities of success.

Notable studies reveal contrasting stakeholder priorities. A UK-based investigation demonstrated that patients, dental students, and practicing dentists emphasize distinct attributes when characterizing a successful dentist, underscoring the subjectivity of these perceptions [1]. Separate research on UK vocational trainers identified technical proficiency as their foremost criterion, with communication skills and diagnostic acumen ranking second and third, respectively [2]. Complementing these findings, a Hong Kong study specifically explored dental students’ views on the hallmarks of an exemplary dentist, adding regional and generational perspectives to the discourse [3].

While professional achievements, reputation, and financial stability are commonly associated with success, the absence of standardized definitions highlights the importance of investigating emerging practitioners’ viewpoints. By analyzing how students conceptualize dental success, educators and policymakers can better align training programs with evolving professional standards, ultimately bridging the gap between academic preparation and real-world practice demands.

Previous research has highlighted the importance of technical competence [4], communication skills [5], ethical behavior [6], and patient-centered care [7] in establishing a successful dental practice. However, there is limited data specifically exploring how dental students perceive these attributes and what qualities they deem most critical for success in dentistry [3]. Gaining insights into their perspectives can inform curriculum development and mentorship programs aimed at cultivating well-rounded future dentists.

Clinical expertise, including clinical competence and experience, is generally considered a crucial attribute for enhancing the quality of dental care and fostering patient trust and satisfaction [8]. Clinical competence refers to a dentist’s ability to perform dental procedures accurately and effectively, which ensures that patients receive high-quality care that leads to better health outcomes and reduced complications [9]. This competence is essential for ensuring patient safety, as diagnostic errors are often associated with gaps in knowledge and clinical experience [10]. Developing strong diagnostic skills helps reduce errors and prevent adverse events that could compromise patient trust. Furthermore, robust diagnostic abilities are a fundamental aspect of clinical competence, allowing dentists to accurately evaluate conditions and recommend suitable treatment plans—both of which are crucial for effective patient management.

In addition, clinical experience provides dentists with the practical knowledge necessary to apply theoretical concepts in real-world settings [3]. This hands-on experience is invaluable for honing skills and building confidence, allowing dentists to encounter a variety of cases that enhance their problem-solving abilities. Such experience equips them to think critically and make informed decisions quickly, and this skill is especially important in emergency situations. Additionally, clinical experience fosters stronger communication and interpersonal skills, as dentists learn how to engage effectively with patients, address their concerns, and provide reassurance, all of which contribute significantly to patient satisfaction [11,12]. Each patient interaction serves as a learning opportunity, allowing dentists to refine their skills and stay updated on best practices.

The role of a dentist extends far beyond mere clinical expertise; it encompasses a range of essential attributes that are crucial for effective practice. Key among these are effective communication, empathy, ethical practice, and leadership skills [13]. Effective communication ensures that dentists can clearly convey treatment options, address patient concerns, and foster a supportive environment, which is fundamental for building trust [14]. Empathy allows dentists to understand and respond to the emotional and psychological needs of their patients, contributing to a more compassionate care experience [15]. Additionally, ethical practice is paramount in maintaining professional integrity and ensuring that patient welfare is always prioritized [16]. Leadership skills are equally important, as they enable dentists to guide their teams, influence patient care practices, and contribute positively to the healthcare community [17].

As dental education evolves to meet the complexities of modern healthcare, understanding the perspectives of dental students regarding what constitutes a good and successful dentist becomes increasingly important. Insight into these perspectives can illuminate the qualities that future dentists value most, informing curriculum development and teaching methodologies. By integrating these attributes into training programs, dental schools can cultivate well-rounded professionals who are not only technically proficient but also adept in interpersonal and ethical dimensions of care [18]. This holistic approach to dental education can ultimately enhance the quality of patient care. Moreover, it ensures that graduates are better equipped to navigate the challenges of contemporary dental practice, contributing to public health outcomes.

While studies have explored the qualities of a successful dentist in various contexts, a gap remains regarding the perspectives of dental students in mainland China. Previous research, including a similar study conducted in Hong Kong, China, has highlighted attributes like clinical competence, good communication skills, and responsibility [3]. However, these qualities are often shaped by cultural, educational, and societal factors [19,20]. The unique socio-cultural environment of mainland China, the emphasis on traditional values, the structure of dental education, and patient expectations could lead to distinct perspectives among dental students in China regarding what defines a successful dental practice. Given the socio-cultural, educational, and healthcare system differences between Hong Kong and mainland China, this study hypothesizes that dental students in mainland China will hold distinct perspectives regarding the essential qualities of a good and successful dentist compared to their counterparts in Hong Kong. This study aims to explore the perspectives of dental students at a university in mainland China regarding the attributes that define a good or successful dentist.

## 2. Methods

A cross-sectional survey on the essential attributes of a good and successful dentist was conducted at The School of Stomatology, Fujian Medical University (FMUSS) from March 2025 to August 2025. The study protocol was approved by the local Institutional Review Board. Informed consent was obtained from all participants. Participants had to sign the informed consent at the beginning of the electronic questionnaire survey before moving on to answer the first question. FMUSS offers a 5-year dental degree program with Chinese as the medium of instruction. All 651 dental students were invited to participate, with no exclusion criteria applied.

Two trained research assistants conducted the survey immediately after the students attended their scheduled lectures in lecture halls or participated in practical sessions in the simulation laboratory. The survey was carried out with prior approval obtained from the respective instructors or course coordinators to ensure institutional compliance. All participating students were provided with a hyperlink to an electronic questionnaire survey, which they completed voluntarily on the spot using their personal devices. The research assistants collected all completed questionnaires after a 15 min data collection window to ensure timely and efficient data gathering for subsequent analysis.

This study utilized a structured questionnaire from a previous study, which was designed to assess dental students’ perspectives on essential qualities for dental practice in Hong Kong. The original questionnaire was developed in English, and the initial translation into Chinese was carried out using the forward-backward translation method [21]. Two bilingual experts, each proficient in both languages and familiar with the subject matter, independently translated the questionnaire into Chinese. The two Chinese translations were compared and synthesized into a single preliminary version through discussion among the translators and a bilingual researcher. Discrepancies were resolved through consensus, ensuring that the translated items retained the meaning of the original.

The reconciled Chinese version was then independently back-translated into English by two different bilingual individuals who were blinded to the original questionnaire. These translators had no prior exposure to the original instrument to prevent bias. The pre-final Chinese version was pilot-tested with three native Chinese speakers to assess clarity and cultural appropriateness. Feedback from this pre-test was incorporated to refine the questionnaire. The reconciled Chinese version was then independently backtranslated into English by two different bilingual individuals who were blinded to the original questionnaire. These translators had no prior exposure to the original instrument to prevent bias. The pre-final Chinese version was pilot-tested with two native Chinese speakers to assess clarity and cultural appropriateness. Feedback from this pre-test was incorporated to refine the questionnaire.

**Figure 1:**
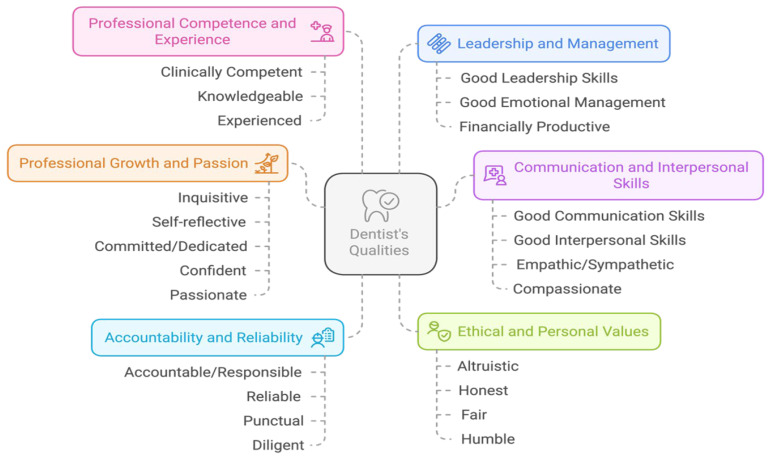
The six categories and qualities of dentist (adapted from Ho et al., 2025 [3]).

The questionnaire survey consisted of four questions with the same 23 predefined qualities as options and an option of other with a blank for participants to fill in. Students were asked to identify the three most important qualities for each question. Demographic information, such as sex, age, and year of study, was collected. The first two questions explored the qualities that dental students associate with “a successful dentist” and “a good dentist”, respectively, without providing any specific definitions or criteria for these terms. The third question focused on the qualities students expect their dentists to possess, while the fourth question examined the qualities that should be emphasized in dental school training. Thematic analysis was performed in our previous study to categorize the qualities [3]. The six categories of the dentist’s qualities were developed through this process, as shown in Figure 1.

During data analysis, chi-square tests were used to compare response patterns by sex and academic seniority (year of study). Bivariate analyses focused on attributes selected as “key qualities” by at least 15% of participants. These analyses were stratified by sex and year of study to identify group-specific trends. The chi-square test evaluated associations between categorical variables, while Cochran’s Q test assessed whether the perceived importance of key dentist attributes differed significantly across the four survey questions. Fisher’s exact test was performed when 20% of cells had an expected count of less than 5. All analyses were performed using SPSS (version 29), with statistical significance defined as *p* < 0.05.

## 3. Results

A total of 651 students, from BDS 1 to 5, were invited to participate in the survey. Their age ranged from 18 to 24 years, with a mean (standard deviation) age of 20 (2). A total of 645 complete questionnaires were collected, yielding a response rate of 99% (645/651).

The primary reason for non-response was absence from class at the time of data collection. Among the 645 students, 281 (44%) were male and 364 were female. All questionnaires collected were valid, except for three students who did not respond to the demographic questions regarding their years of study.

Table 1 highlights the five most important qualities students rated for a successful dentist and a good dentist, qualities expected from their dentists when they are patients, and the five most important qualities that students think dental schools should focus on when training dentists.

Among the qualities, “clinical competence” was ranked as the most important quality across all four categories, with 531 (83%) responses for a successful dentist, 467 (72%) for a good dentist, 498 (76%) for expected qualities from their dentists, and 432 (66%) for qualities that dental training should focus on. Other highly endorsed qualities included “experienced”, “knowledgeable”, “good communication skills”, and “accountable/responsible”. The qualities that were least endorsed were “punctual” and “altruism”.

**Figure 2:**
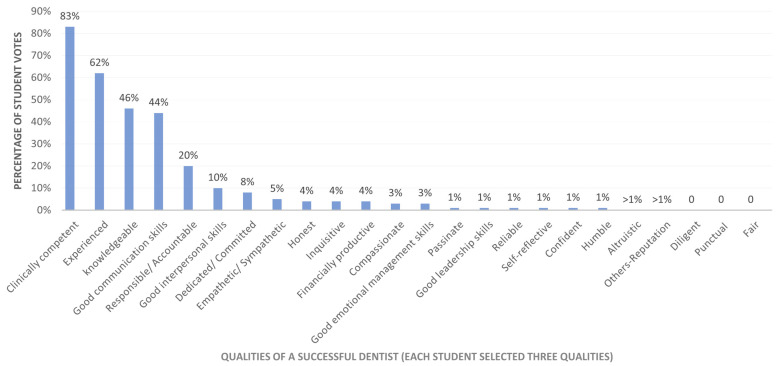
Distribution of essential qualities of a successful dentist as ranked by dental students (*n* = 642).

Figure 2 presents the students’ rankings of the three most important qualities of a successful dentist. Clinical competence (83%, *n* = 531), experienced (62%, *n* = 401), knowledgeable (46%, *n* = 296), good communication skills (44%, *n* = 280), and a sense of responsibility or accountability (20%, *n* = 129) were ranked as the top five attributes by dental students.

**Figure 3:**
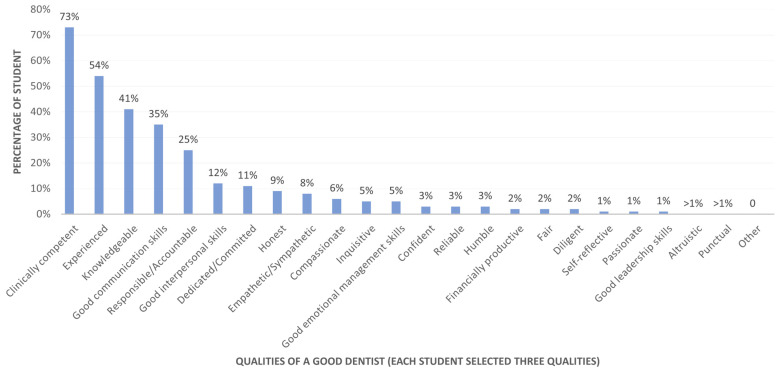
Distribution of essential qualities of a good dentist as ranked by dental students (*n* = 642).

Figure 3 presents the students’ rankings of the three most important qualities of a good dentist. Clinical competence (78%, *n* = 467), experienced (67%, *n* = 345), knowledgeable (38%, *n* = 262), good communication skills (34%, *n* = 226), and a sense of responsibility or accountability (22%, *n* = 163) were again ranked as the top five attributes by dental students.

**Figure 4:**
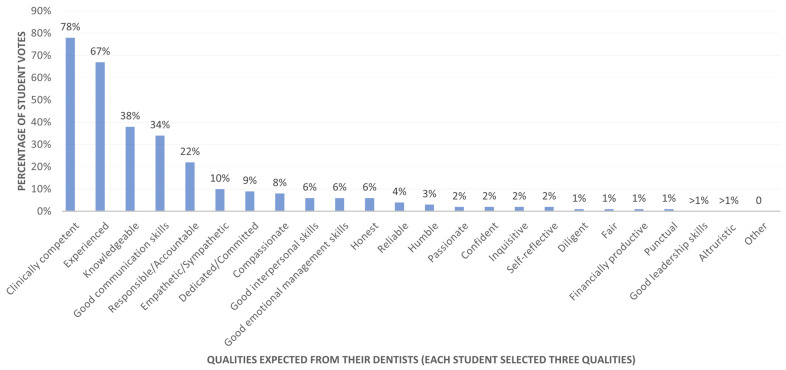
Student rankings of essential qualities expected from their dentists (*n* = 642).

Figure 4 illustrates the students’ perceptions of the qualities they expect from a dentist when they are patients. Clinical competence (78%, *n* = 498), experienced (67%, *n* = 431), knowledgeable (38%, *n* = 241), good communication skills (34%, *n* = 219), and a sense of responsibility or accountability (22%, *n* = 141) were again ranked as the top five attributes by dental students.

**Figure 5:**
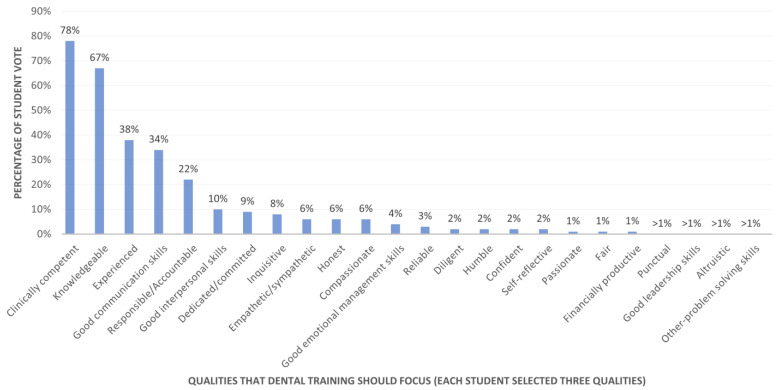
Student rankings of key qualities for university dental education focus (*n* = 642).

Lastly, Figure 5 shows the students’ rankings of the qualities that should be emphasized in university education for dental students. Clinical competence (67%, *n* = 432), knowledgeable (49%, *n* = 316), experienced (49%, *n* = 315), good communication skills (31%, *n* = 202), and a sense of responsibility or accountability (27%, *n* = 173) were ranked as the top five attributes by dental students.

Table 2 and Table 3 display the essential qualities identified by students, categorized by sex and year of study, for a successful dentist and a good dentist, respectively.

Table 4 presents the students’ rankings of the qualities they expect from a dentist when they are patients. Additionally, Table 5 outlines the qualities that university education should emphasize for dental students, broken down by students’ sex and year of study.

Bivariate analysis was conducted for key qualities selected by at least 15% of the students, stratified by sex and year of study (Table 6). A significant difference was observed in the ranking of “good communication skills” between junior and senior students across all four items. More senior students (Year 4–5) than junior students (Year 1–3) considered good communication skills an important quality for a successful dentist (*p* < 0.001), a good dentist (*p* = 0.002), the dentist they visited (*p* = 0.01), and for dental education to focus on (*p* = 0.02). Male students were more likely than female students to regard knowledge as an important attribute of a successful dentist (*p* = 0.002) and a good dentist (*p* = 0.006), as well as an important quality expected from their dentist when they are patients (*p* = 0.001).

Cochran’s Q test was performed to compare the frequencies of major dentist qualities across the four questions (Table 7). The results revealed significant differences (*p* < 0.001) in how dental students prioritized qualities across the four questions. “Clinical competence” was most frequently selected for a successful dentist (Q1, 82%) and their own dentist (Q3, 77%), but less so for dental training focus (Q4, 67%). “Knowledgeable” was linked to a successful dentist (Q1, 46%) and dental training focus (Q4, 49%) more than their own dentist (Q3, 37%). “Experienced” was prioritized for a successful dentist (Q2, 62%) and their own dentist (Q3, 67%), while less so for a good dentist (Q2, 54%) and for dental training (Q4, 49%). “Good communication skills” (Q1, 43%) and “accountable/responsible” (Q1, 20%) showed context-dependent prioritization.

**Figure 6:**
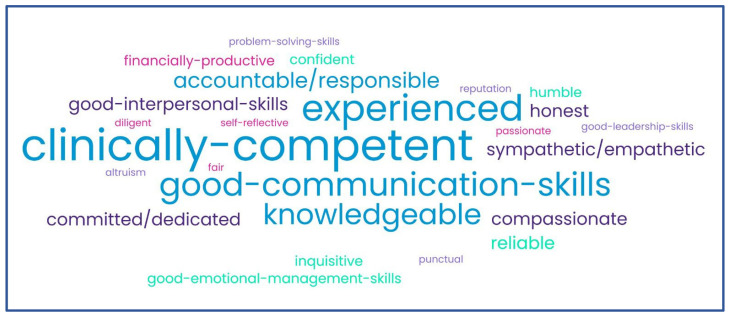
Students’ perspectives on essential attributes of a successful and good dentist.

A word cloud is shown in Figure 6. It visually summarizes the results of the students’ perspectives on the essential attributes of good and successful dentists, making it easier to identify at a glance important words and themes.

## 4. Discussion

Dentistry requires a combination of skills and qualities to provide high-quality patient care and achieve professional success. This study investigated the perspectives of dental students in China regarding the essential qualities of a good dental practice. The results were to be compared with those in our previous study carried out in Hong Kong [3]. No explanations or definitions of the terms such as “good dentist” or “successful dentist were given to students prior to survey distribution to maintain data integrity and obtain authentic perceptions.

By building upon prior research conducted in Hong Kong, this study examined these perceptions, providing insights that can inform tailored educational strategies and professional development programs within China. The findings contribute to a better understanding of the cultural and contextual factors influencing dental students’ views on professionalism and competence in the Chinese setting. The results provide valuable insights into mainland Chinese dental students’ perceptions of the attributes that define a successful dental practice and enable comparison of these perspectives with those of Chinese students in Hong Kong [3].

The consistent emphasis on clinical competence, experience, and knowledge across all four survey questions underscores the centrality of technical proficiency in the students’ conceptualization of a successful dental practitioner. This aligns with the global understanding of dental professionalism, where clinical skills form the foundation of effective practice. However, the study also highlights nuanced differences in how students perceive the importance of various qualities depending on context—whether defining success, evaluating their own dentists, or considering what should be emphasized in education.

### 4.1. Clinical Competence and Experience as Cornerstones

The preeminent ranking of clinical competence and experience among the students aligns with the fundamental nature of dentistry as a technical discipline. The students’ recognition of these attributes reflects the importance they place on the ability to perform procedures accurately and effectively, which directly impacts patient safety, treatment outcomes, and overall trust [22,23]. The high endorsement rate (83% for a successful dentist, 72% for a good dentist, etc.) indicates a shared understanding that mastery of clinical skills is essential for success in dental practice. Moreover, the emphasis on experience suggests that students value practical, hands-on knowledge, which they believe can be acquired through clinical exposure. This aligns with the broader literature emphasizing that clinical experience enhances problem-solving skills, critical thinking, diagnostic skills, and confidence [24,25,26]—all vital for effective patient management. It also highlights the importance of experiential learning in dental education curricula, where early and continuous clinical exposure can bridge the gap between theoretical knowledge and real-world application [27].

### 4.2. Communication Skills and Responsibility

While technical skills are paramount, the study also underscores the significance of communication skills and a sense of responsibility and accountability. These attributes, ranked highly across all questions, reflect an evolving recognition among students that effective dentists must also excel in interpersonal domains. Good communication skills are critical for building rapport, explaining procedures, and managing patient expectations [28,29]. The fact that senior students (Years 4–5) considered communication skills more important than juniors suggests a developing awareness of the relational aspects of dental practice, possibly gained through clinical experience and patient interactions over time. These results contrast with those of our previous study conducted in Hong Kong, where both senior and junior dental students regarded communication skills as equally important in dental practice [3].

In the earlier study, no significant difference was observed between the two groups in their perception of the importance of communication skills [3]. This divergence may suggest cultural or educational differences that influence students’ perceptions of professional competencies at different stages of their training. Further investigation is needed to understand the underlying factors contributing to these differing attitudes toward communication skills across different contexts. Responsibility/accountability are essential for maintaining professional integrity and trustworthiness. The high ranking of these qualities indicates that students associate success with not only technical proficiency but also ethical and responsible behavior. This holistic view of professionalism is consistent with modern dental education paradigms, which emphasize ethical practice, patient-centered care, and leadership [30,31].

### 4.3. Other Attributes for Success or Good Dentist

More senior students than junior students valued the importance placed on communication skills for successful or good dentists. As students progress through their training and gain more patient interaction experiences, they might recognize the critical role that communication plays in successful practice [29,32]. Such developmental shifts underscore the importance of integrating communication training early and consistently in dental curricula, with reinforcement throughout clinical education [33].

Additionally, attributes such as punctuality and altruism received the lowest ratings. This may reflect cultural perceptions within the context of Chinese dental education and practice, where these qualities, although still important, are perhaps viewed as basic professional manners rather than defining characteristics of a successful dentist. Alternatively, students may prioritize attributes directly linked to clinical performance and patient outcomes over personal virtues perceived as secondary.

### 4.4. Implications for Dental Education

These findings have several implications for dental education in China. First, curricula should prioritize developing clinical competence and practical skills, ensuring students gain ample hands-on experience. Second, communication training should be emphasized and integrated into clinical courses [33], promoting interpersonal skills alongside technical proficiency. Third, fostering a sense of responsibility and ethical practice should be a core component, potentially through case-based discussions and professionalism modules [34]. Furthermore, the relatively low ratings for attributes like punctuality and altruism do not diminish their importance but suggest they may be considered baseline professional behaviors. Educational strategies should reinforce these virtues, framing them as integral to professional identity and patient trust.

### 4.5. Socio-Cultural Context and Broader Significance

It is also important to consider the socio-cultural context that influences students’ perceptions of the ideal attributes of a dentist [35]. In China, traditional values emphasizing technical mastery and a strong sense of responsibility likely shape students’ priorities regarding the essential qualities of a good dental practice. When comparing these findings to our previous study conducted in Hong Kong [3], we observed that students in Hong Kong ranked sympathy and empathy as important qualities in a good dentist, whereas students in China did not regard these traits as highly. This difference may reflect Hong Kong students’ greater emphasis on a holistic, patient-centered approach to care and the importance of the dentist-patient relationship. At the same time, the increasing emphasis on communication skills among senior students in China demonstrates a positive and progressive shift towards valuing interpersonal skills in dental practice. This trend aligns well with global developments in healthcare professionalism and highlights the evolving understanding of comprehensive patient care [36,37]. Recognizing these socio-cultural nuances is vital for educators, as it supports the development of curricula that effectively combine technical excellence with interpersonal and ethical qualities, ultimately fostering well-rounded and socio-culturally sensitive dental professionals.

### 4.6. Limitations and Future Directions

While this study provides valuable insights, it is limited to students from a single university in mainland China. Hence, this study does not represent broader national or regional perspectives. Future research could explore practicing dentists’ views, patient expectations, and comparisons across different cultural or educational settings. Longitudinal studies tracking changes in perceptions over time could also elucidate how educational experiences influence the prioritization of various attributes. Additionally, the survey design may introduce bias by requiring students to select the “most important” characteristics of a successful dentist and a good dentist. Given that clinical competence and experience are central goals of dental education, students are likely to prioritize these traits over others, such as reliability. The lower ranking of traits like “reliable” may not indicate that students undervalue them. The survey’s forced-choice format simplified the survey’s efficiency. However, it provides limited insight into whether unselected traits are perceived as less important or simply foundational.

## 5. Conclusions

This study confirms that mainland Chinese dental students view clinical competence, experience, knowledge, communication skills, and responsibility as the essential attributes of a successful dentist. These findings reinforce the importance of comprehensive dental education that integrates technical skills development with interpersonal and ethical training. Recognizing and cultivating these attributes will not only prepare students for effective practice but also enhance patient care quality and professional satisfaction. As dental education continues to evolve, incorporating student perceptions into curriculum design will ensure that future dentists are equipped with the multifaceted competencies required for success in modern healthcare environments.

## Figures and Tables

**Table 1:** Essential qualities of dentists by student votes.

Key Dentist Qualities	For a Successful Dentist	For a Good Dentist	Of the Student’s Own Dentist	That Dental Training Should Focus
Clinically competent	**531**	**467**	**498**	**432**
Experienced	**401**	**345**	**431**	**315**
Knowledgeable	**296**	**262**	**241**	**316**
Good communication skills	**280**	**226**	**219**	**202**
Accountable/Responsible	**129**	**163**	**141**	**173**
Good interpersonal skills	58	77	39	65
Committed/Dedicated	51	69	56	60
Sympathetic/Empathetic	32	51	61	55
Honest	28	55	38	45
Inquisitive	26	34	12	58
Compassionate	22	39	50	40
Good emotional management	22	33	39	29
Financially productive	23	13	5	7
Reliable	6	18	23	28
Humble	4	17	17	15
Confident	4	20	14	15
Passionate	8	9	15	14
Self-reflective	6	9	10	15
Good leadership skills	7	4	3	5
Diligent	0	10	9	21
Fair	0	10	7	14
Punctual	0	1	4	6
Altruism	1	3	3	3
Others	1	0	0	1
(Please specify)	(Reputation)			(Problem-solving skills)

Those bolded are the top five most highly rated qualities.

**Table 2:** Essential Qualities of a Successful Dentist Rated by Students based on Sex and Year of Study.

Essential Qualities for a Successful Dentist	Male (*n* = 281)	Female (*n* = 364)	Total (*n* = 645)	*p*-Value	Year 1–3 (*n* = 420)	Year 4–5 (*n* = 222)	Total (*n* = 642)	*p*-Value
*Professional Competence & Experience*								
Clinical Competence	233 (83%)	298 (82%)	531 (82%)	0.73	337 (80%)	192 (87%)	529 (82%)	**0.049**
Knowledgeable	148 (53%)	147 (40%)	296(46%)	**0.002**	190 (45%)	104 (47%)	294 (46%)	0.700
Experienced	171 (61%)	230 (63%)	401 (62%)	0.545	262 (62%)	137 (62%)	399 (62%)	0.870
*Communication & Interpersonal Skills*								
Good communication skills	114 (41%)	166 (46%)	280 (43%)	0.201	165 (39%)	114 (51%)	279 (44%)	**0.003**
Good interpersonal skills	25 (9%)	33 (9%)	58 (9%)	0.941	43 (10%)	14 (6%)	57 (9%)	0.810
Empathic/Sympathetic	15 (5%)	17 (5%)	32 (5%)	0.699	19 (5%)	13 (6%)	32 (5%)	0.461
Compassionate	7 (3%)	15 (4%)	22 (3%)	0.258	15 (4%)	7 (3%)	22 (3%)	0.189
*Accountability & Reliability*								
Accountable/responsible	46 (16%)	83 (23%)	129 (20%)	**0.045**	96 (23%)	33 (15%)	129(20%)	**0.016**
Reliable	3 (1%)	3 (1%)	6 (1%)	1.000 #	2 (1%)	4 (2%)	6 (1%)	0.782
Diligent	0	0	0	N/A	0	0	0	N/A
Punctual	0	0	0	N/A	0	0	0	N/A
*Leadership & Management*								
Financially productive	16 (6%)	7 (2%)	23 (4%)	**0.01**	14 (3%)	8 (4%)	22 (3%)	0.858
Good leadership skills	2 (1%)	5 (1%)	7 (1%)	0.705 #	4 (1%)	2 (1%)	6 (1%)	1.000 #
Good emotional management	10 (4%)	12 (3%)	22 (3%)	0.856	14 (3%)	8 (4%)	22 (3%)	0.858
*Professional Growth & Passion*								
Inquisitive	8 (3%)	18 (5%)	26 (4%)	0.179	20 (3%)	6 (3%)	26 (4%)	0.208
Confident	2 (1%)	2 (1%)	4 (1%)	1.000	2 (1%)	2(1%)	4 (1%)	0.612 #
Self-reflective	2 (1%)	4 (1%)	6 (1%)	0.701 #	4 (1%)	2 (1%)	6 (1%)	1.000 #
Committed/dedicated	21 (8%)	28 (8%)	49 (8%)	0.917	39 (9%)	10 (5%)	49 (8%)	**0.030**
Passionate	3 (1%)	5 (1%)	8 (1%)	1.000 #	6 (1%)	2 (1%)	8 (1%)	0.721 #
*Ethical Values & Personal Values*								
Honest	13 (5%)	15 (4%)	28 (4%)	0.755	20 (5%)	8 (4%)	28 (4%)	0.494
Humble	3 (1%)	1 (>1%)	4 (1%)	0.323 #	4 (1%)	0 (0)	4 (1%)	0.304 #
Altruistic	0 (0)	1 (>1%)	2 (>1%)	1.000 #	1 (>1%)	0 (0)	1 (>1%)	1.000 #
Fair	0	0	0	N/A	0	0	0	N/A

# Fisher’s exact test was performed when 20% of cells had an expected count less than 5.

**Table 3:** Essential Qualities of a Good Dentist Rated by Students Based on Sex and Year of Study.

Essential Qualities for a Successful Dentist	Male (*n* = 281)	Female (*n* = 364)	Total (*n* = 645)	*p*-Value	Year 1–3 (*n* = 420)	Year 4–5 (*n* = 222)	Total (*n* = 642)	*p*-Value
*Professional Competence & Experience*								
Clinical Competence	209 (74%)	258 (71%)	467 (72%)	0.324	298 (71%)	167(75%)	485 (72%)	0.250
Knowledgeable	131 (47%)	131 (36%)	262 (41%)	**0.006**	174 (41%)	86 (39%)	260 (41%)	0.510
Experienced	152 (54%)	193 (53%)	345 (54%)	0.787	217 (52%)	126 (57%)	343 (53%)	0.220
*Communication & Interpersonal Skills*								
Good communication skills	99 (35%)	127 (35%)	226 (35%)	0.928	130 (31%)	96 (43%)	226 (35%)	**0.002**
Good interpersonal skills	40 (14%)	37 (10%)	77 (12%)	0.114	49 (12%)	27 (12%)	76 (12%)	0.853
Empathic/Sympathetic	24 (9%)	27 (7%)	51 (8%)	0.600	28 (7%)	22 (10%)	50 (8%)	0.145
Compassionate	17 (6%)	22 (6%)	39 (6%)	0.998	31 (7%)	8 (4%)	39 (6%)	0.057
*Accountability & Reliability*								
Accountable/responsible	55 (20%)	96 (26%)	151 (23%)	**0.043**	108 (26%)	43 (20%)	151 (24%)	0.070
Reliable	6 (2%)	12 (3%)	18 (3%)	0.375	13 (3%)	5 (2%)	18 (3%)	0.538
Diligent	3 (1%)	7 (2%)	10 (2%)	0.526 #	7 (2%)	3 (1%)	10 (2%)	1.000 #
Punctual	0 (0)	1 (>1%)	1 (>1%)	1.000 #	1 (>1%)	0 (0)	1 (>1%)	1.000 #
*Leadership & Management*								
Financially productive	7 (3%)	6 (2%)	13 (2%)	0.45	7 (2%)	5 (2%)	12 (2%)	0.760 #
Good leadership skills	3 (1%)	1 (>1%)	4 (1%)	0.323 #	1 (>1%)	3 (1%)	4 (1%)	0.122 #
Good emotional management	12 (4%)	21 (6%)	33 (5%)	0.392	24 (6%)	9 (4%)	33 (5%)	0.365
*Professional Growth & Passion*								
Inquisitive	12 (4%)	22 (6%)	34 (5%)	0.318	29 (7%)	5 (2%)	34 (5%)	0.120
Confident	11 (4%)	9 (3%)	20 (3%)	0.295	13 (3%)	7 (3%)	20 (3%)	0.968
Self-reflective	2 (1%)	7 (2%)	9 (1%)	0.312 #	5 (1%)	4 (2%)	9 (1%)	0.505 #
Committed/dedicated	19 (7%)	46 (13%)	65 (10%)	**0.014**	50 (12%)	15 (7%)	65 (10%)	**0.040**
Passionate	2 (1%)	7 (2%)	9 (1%)	1.000 #	6 (1%)	3 (1%)	9 (1%)	1.000 #
*Ethical Values & Personal Values*								
Honest	27 (10%)	28 (8%)	55 (9%)	0.388	31 (7%)	24 (11%)	55 (9%)	0.140
Humble	7 (3%)	10 (3%)	17 (3%)	0.84	12 (3%)	5 (2%)	17 (3%)	0.650
Altruistic	0 (0)	3 (1%)	3 (1%)	0.261 #	3 (1%)	0 (0)	3 (1%)	0.555 #
Fair	3 (1%)	7 (2%)	10 (2%)	0.526 #	10 (2%)	0 (0)	10 (2%)	0.180 #

# Fisher’s exact test was performed when 20% of cells had an expected count less than 5.

**Table 4:** Essential Qualities Students look for in Their Own Dentists by Sex and Year of Study.

Essential Qualities for a Successful Dentist	Male (*n* = 281)	Female (*n* = 364)	Total (*n* = 645)	*p*-Value	Year 1–3 (*n* = 420)	Year 4–5 (*n* = 222)	Total (*n* = 642)	*p*-Value
*Professional Competence & Experience*								
Clinical Competence	221 (79%)	277 (76%)	498 (77%)	0.44	320 (76%)	175 (79%)	495 (77%)	0.450
Knowledgeable	126 (45%)	115 (32%)	241 (37%)	**<0.001**	145 (35%)	93 (42%)	238 (37%)	0.070
Experienced	184 (66%)	247 (68%)	431 (67%)	0.53	275 (66%)	153 (69%)	428 (67%)	0.380
*Communication & Interpersonal Skills*								
Good communication skills	92 (33%)	127 (35%)	219 (34%)	0.57	129 (31%)	90 (41%)	219 (34%)	**0.010**
Good interpersonal skills	19 (7%)	20 (6%)	39 (6%)	0.503	23 (6%)	16 (7%)	39 (6%)	0.382
Empathic/Sympathetic	25 (9%)	35 (10%)	60 (9%)	0.755	33 (8%)	27 (12%)	60 (9%)	0.750
Compassionate	17 (6%)	33 (9%)	50 (8%)	0.156	33 (8%)	17 (8%)	50 (8%)	0.929
*Accountability & Reliability*								
Accountable/responsible	47 (17%)	90 (25%)	137 (21%)	**0.014**	101 (24%)	36 (16%)	137 (21%)	**0.021**
Reliable	9 (3%)	14 (4%)	23 (4%)	0.662	21 (5%)	2 (1%)	23 (4%)	**0.008**
Diligent	4 (1%)	5 (1%)	9 (1%)	1.000 #	8 (2%)	1 (1%)	9 (1%)	0.174 #
Punctual	2 (1%)	2 (1%)	4 (1%)	1.000 #	4 (1%)	0 (0)	4 (1%)	0.304 #
*Leadership & Management*								
Financially productive	3 (1%)	2 (1%)	5 (1%)	0.658 #	3 (1%)	2 (1%)	5(1%)	1.000 #
Good leadership skills	2 (1%)	1 (>1%)	3 (1%)	0.583 #	2 (1%)	1 (1%)	3 (1%)	1.000 #
Good emotional management	15 (5%)	24 (7%)	39 (6%)	0.507	31 (7%)	8 (4%)	39 (6%)	0.057
*Professional Growth & Passion*								
Inquisitive	6 (2%)	6 (2%)	12 (2%)	0.650	10 (2%)	2 (1%)	12 (2%)	0.234 #
Confident	8 (3%)	6 (2%)	14 (2%)	0.300	8 (2%)	6 (3%)	14 (2%)	0.573 #
Self-reflective	3 (1%)	7 (2%)	10 (2%)	0.526	9 (2%)	1 (1%)	10 (2%)	0.177 #
Committed/dedicated	25 (9%)	29 (8%)	54 (8%)	0.672	43 (10%)	11 (5%)	54 (8%)	**0.022**
Passionate	4 (1%)	11 (3%)	15 (2%)	0.182	14 (3%)	1 (1%)	15 (2%)	**0.021**
*Ethical Values & Personal Values*								
Honest	18 (6%)	20 (6%)	38 (6%)	0.626	23 (6%)	15 (7%)	38 (6%)	0.513
Humble	8 (3%)	9 (3%)	17 (3%)	0.768	11 (3%)	6 (3%)	17 (3%)	0.950
Altruistic	1 (>1%)	2 (1%)	3 (1%)	1.000 #	3 (1%)	0 (0)	3 (1%)	0.555 #
Fair	1 (>1%)	6 (2%)	7 (1%)	0.145 #	5 (1%)	2 (1%)	7 (1%)	1.000 #

# Fisher’s exact test was performed when 20% of cells had an expected count less than 5.

**Table 5:** Essential Qualities for Dental Education Focus as Rated by Students by Sex and Year of Study.

Essential Qualities for a Successful Dentist	Male (*n* = 281)	Female (*n* = 364)	Total (*n* = 645)	*p*-Value	Year 1–3 (*n* = 420)	Year 4–5 (*n* = 222)	Total (*n* = 642)	*p*-Value
*Professional Competence & Experience*								
Clinical Competence	194 (69%)	238 (65%)	432 (67%)	0.33	277 (66%)	153 (69%)	430 (67)	0.450
Knowledgeable	149 (53%)	167 (46%)	316 (49%)	0.72	198 (47%)	116 (52%)	314 (49%)	0.220
Experienced	145 (52%)	170 (47%)	315 (49%)	0.22	199 (47%)	114 (51%)	313 (49%)	0.340
*Communication & Interpersonal Skills*								
Good communication skills	84 (30%)	118 (32%)	202 (30%)	0.49	118 (28%)	83 (37%)	201 (31%)	**0.020**
Good interpersonal skills	31 (11%)	34 (9%)	65 (10%)	0.479	41 (10%)	23 (10%)	64 (10%)	0.810
Empathic/Sympathetic	25 (9%)	28 (7%)	53 (8%)	0.581	35 (8%)	18 (8%)	53 (8%)	0.921
Compassionate	15 (5%)	25 (7%)	40 (6%)	0.424	27 (6%)	13 (6%)	40 (6%)	0.775
*Accountability & Reliability*								
Accountable/responsible	55 (20%)	110 (30%)	165 (26%)	**0.002**	122 (29%)	43 (20%)	165 (26%)	**0.008**
Reliable	11 (4%)	17 (5%)	28 (4%)	0.640	22 (5%)	6 (3%)	28 (4%)	0.135
Diligent	4 (1%)	17 (5%)	21 (3%)	**0.021**	15 (4%)	6 (3%)	21 (3%)	0.556
Punctual	4 (1%)	2 (1%)	6 (1%)	0.412 #	4 (1%)	2 (1%)	6 (1%)	1.000 #
*Leadership & Management*								
Financially productive	4 (1%)	3 (1%)	7 (1%)	0.476 #	5 (1%)	1 (1%)	6 (1%)	0.670 #
Good leadership skills	5 (1%)	2 (1%)	5 (1%)	0.658 #	3 (1%)	2 (1%)	5 (1%)	1.000 #
Good emotional management	14 (5%)	15 (4%)	29 (5%)	0.601	24 (6%)	5 (2%)	29 (5%)	0.450
*Professional Growth & Passion*								
Inquisitive	20 (7%)	38 (10%)	58 (9%)	0.144	34 (8%)	24 (11%)	58 (9%)	0.254
Confident	8 (3%)	7 (2%)	15 (2%)	0.440	10 (2%)	5 (2%)	15 (2%)	0.918
Self-reflective	10 (4%)	5 (1%)	15 (2%)	0.068	9 (2%)	6 (3%)	15 (2%)	0.655
Committed/dedicated	22 (8%)	38 (10%)	60 (9%)	0.258	41 (10%)	19 (9%)	60 (9%)	0.618
Passionate	6 (2%)	8 (2%)	14 (2%)	0.957	10 (2%)	4 (2%)	14 (2%)	0.780
*Ethical Values & Personal Values*								
Honest	21 (7%)	24 (7%)	45 (7%)	0.664	30 (7%)	15 (7%)	45 (7%)	0.855
Humble	11 (4%)	5 (1%)	16 (3%)	**0.04**	13 (3%)	3 (1%)	16 (3%)	0.178
Altruistic	0 (0)	3 (1%)	3 (1%)	0.261 #	3 (1%)	0 (0)	3 (1%)	0.555 #
Fair	4 (1%)	10 (3%)	14 (2%)	0.253	11 (3%)	3 (1%)	14 (2%)	0.400 #

# Fisher’s exact test was performed when 20% of cells had an expected count less than 5.

**Table 6:** Bivariate Analysis of Major Dentist Qualities Rated by Students, by Sex and Year of Study.

Dentist Qualities Rated by Students	Sex (*n* = 645)		*p* Value	Year of Study (*n* = 642)		*p* Value
	Male (*n* = 281)	Female (*n* = 364)		Year 1–3 (*n* = 420)	Year 4–5 (*n* = 222)	
Dentist qualities for a successful dentist						
Clinical Competence	233 (63%)	298 (82%)	0.73	337 (80%)	192 (87%)	**0.** **049**
Knowledgeable	148 (53%)	147 (40%)	**0.** **002**	190 (45%)	104 (47%)	0.70
Experienced	171 (61%)	230 (63%)	0.545	262 (62%)	137 (62%)	0.87
Good communication skills	171 (61%)	166 (46%)	0.201	165 (39%)	114 (51%)	**0.** **003**
Accountable/Responsible	46 (16%)	83 (23%)	**0.045**	96 (23%)	33 (15%)	**0.016**
Dentist qualities for a good dentist						
Clinical Competence	209 (74%)	258 (71%)	0.324	298 (71%)	167 (75%)	0.25
Knowledgeable	131 (47%)	131 (36%)	**0** **.006**	147 (41%)	86 (39%)	0.51
Experienced	153 (54%)	193 (53%)	0.787	217 (52%)	126 (57%)	0.22
Good communication skills	99 (35%)	127 (35%)	0.928	130 (31%)	96 (43%)	**0.** **002**
Accountable/Responsible	55 (20%)	96 (26%)	**0.043**	108 (26%)	43 (20%)	0.07
Dentist qualities of the students own dentist						
Clinical Competence	221 (79%)	277 (76%)	0.44	320 (76%)	175 (79%)	0.45
Good communication skills	92 (33%)	127 (65%)	0.57	129 (31%)	90 (41%)	**0.** **01**
Experienced	184 (66%)	247 (68%)	0.53	275 (66%)	153 (69%)	0.38
Accountable/responsible	47 (17%)	90 (25%)	**0.** **014**	101 (24%)	36 (16%)	**0.** **021**
Knowledgeable	126 (45%)	115 (32%)	**<0.001**	145 (35%)	93 (42%)	0.07
Dentist qualities that dental training should focus						
Clinical Competence	194 (69%)	238 (65%)	0.33	277 (66%)	153 (69%)	0.45
Knowledgeable	107 (72%)	178 (74%)	0.72	198 (47%)	116 (52%)	0.22
Good communication skills	84 (30%)	118 (32%)	0.49	118 (28%)	83 (37%)	**0.** **02**
Accountable/responsible	55 (20%)	110 (30%)	**0.** **002**	122 (29%)	43 (20%)	**0.0** **08**
Experienced	145 (52%)	170 (47%)	0.22	199 (47%)	114 (51%)	0.34

Bold: *p* ≤ 0.05, significance.

**Table 7:** Comparison of major dentist qualities among four questions.

	Yes (%)	*p* Value	Pairwise Comparisons
*Clinical competence (n = 645)*		*<0.001*	
Q1—for a successful dentist	531 (82%)		Q1 = Q3 Q2 = Q3
Q2—for a good dentist	467 (72%)		
Q3—of the students’ own dentists	498 (77%)		
Q4—that dental training should focus on	432 (67%)		
*Knowledgeable (n =* *645* *)*		*<0.001*	
Q1—for a successful dentist	296 (46%)		Q1 = Q4 Q2 = Q3 Q1 = Q2
Q2—for a good dentist	262 (41%)		
Q3—of the students’ own dentists	241 (37%)		
Q4—that dental training should focus on	316 (49%)		
*Experienced (n =* *645* *)*		*<0.001*	
Q1—for a successful dentist	401 (62%)		Q1 = Q3 Q2 = Q4
Q2—for a good dentist	345 (54%)		
Q3—of the students’ own dentists	431 (67%)		
Q4—that dental training should focus on	315 (49%)		
*Good communication skills (n =* *645* *)*		*<0.001*	
Q1—for a successful dentist	280 (43%)		Q2 = Q3 = Q4
Q2—for a good dentist	226 (35%)		
Q3—of the students’ own dentists	219 (34%)		
Q4—that dental training should focus on	202 (30%)		
*Accountable/responsible (n =* *645* *)*		*<0.001*	
Q1—for a successful dentist	129 (20%)		Q2 = Q3 = Q4
Q2—for a good dentist	151 (23%)		
Q3—of the students’ own dentists	137 (21%)		
Q4—that dental training should focus on	165 (26%)		

## Data Availability

The original contributions presented in this study are included in the article. Further inquiries can be directed to the corresponding authors.
